# Biomechanical comparison of different combinations of hook and screw in one spine motion unit - an experiment in porcine model

**DOI:** 10.1186/1471-2474-15-197

**Published:** 2014-06-09

**Authors:** Ching-Lung Tai, Li-Huei Chen, De-Mei Lee, Mu-Yi Liu, Po-Liang Lai

**Affiliations:** 1 Graduate Institute of Medical Mechatronics, Department of Mechanical Engineering, Chang Gung University, Taoyuan, Taiwan; 2 Department of Orthopaedic Surgery, Chang Gung Memorial Hospital, Chang Gung University College of Medicine, Taoyuan, Taiwan

**Keywords:** Pedicle screw, Lamina hook, Biomechanical study, Porcine model

## Abstract

**Background:**

The biomechanical performance of the hooks and screws in spinal posterior instrumentation is not well-characterized. Screw-bone interface failure at the uppermost and lowermost vertebrae is not uncommon. Some have advocated for the use of supplement hooks to prevent screw loosening. However, studies describing methods for combined hook and screw systems that fully address the benefits of these systems are lacking. Thus, the choice of which implant to use in a given case is often based solely on a surgeon’s experience instead of on the biomechanical features and advantages of each device.

**Methods:**

We conducted a biomechanical comparison of devices instrumented with different combinations of hooks and screws. Thirty-six fresh low thoracic porcine spines were assigned to three groups (12 per group) according to the configuration used for of fixation: (1) pedicle screw; (2) lamina hook and (3) combination of pedicle screw and lamina hook. Axial pullout tests backward on transverse plane in the direction normal to the rods were performed using a material testing machine and a specially designed grip with self-aligned function.

**Results:**

The pullout force for the pedicle screws group was significantly greater than for the hooks and the combination (p < 0.05). However, no significant difference was found between the hooks and the combination (p > 0.05).

**Conclusions:**

Pedicle screws achieve the maximal pullout strength for spinal posterior instrumentation.

## Background

The implantation of bone screws into the pedicle was reported in the early 1960s [1]. Pedicle screw fixation is regarded as a significant improvement over conventional methods for stabilizing the spine, which use wires, hooks or both. Pedicle screws provide three-column fixation, allowing shorter segment of instrumentation to be used to restore sagittal alignment. Despite these advantages, pedicle screws have drawbacks. For example, pedicle screws can bend or break particularly in the caudal segments [2,3]. There have been no clear biomechanical comparisons between hooks and screws. Although pedicle screw anchors offer three-dimensional correction, which is beneficial compared with hooks, pullout tests have shown that the purchase power of a single pedicle screw is greater than a single hook in healthy bone, but less powerful than a single hook in osteoporotic bone [4]. In osteoporotic vertebrae, the bone quality of the lamina is better than the bone quality in the vertebral body. To provide better curve correction and maintain a balanced spine, the anchoring of the implant to the bone should be very strong and resistant to loading.

Harrington’s rod for the treatment of scoliosis was implemented in 1962 as the first implant for thoracic spine surgery [5,6]. This device is a non-segmental hook and rod system. The first attempt to provide segmental fixation was Luque’s sublaminar wire [7,8]. Sublaminar wires provide stable fixation for each vertebral segment but do not reduce coronal spinal deformities. The development of the multiple segmental hook and rod system by Cotrel and Dubousset was a milestone in scoliosis surgery [9,10]. There are two types of hooks that used in this instrumentation: pedicle and lamina. Pedicle hooks provide stronger anchoring power and do not enter the neural foramen. However, they can only translate the correction force upward (cranially) in the thoracic spine. Lamina hooks can provide upward and downward (caudal) forces in the thoracic and lumbar spine. However, they must enter the neural canal to anchor to the lamina. Suk began using thoracic pedicle screws for scoliosis correction in 1991 and has since reported the highest curve correctivity of any system [11]. In spite of this encouraging result, the use of thoracic screws for scoliosis has not been widely accepted. Screw-bone interface failure at the uppermost and lowermost vertebrae is not uncommon. Some have advocated for the use of supplement hooks to prevent screw loosening [11-13]. However, the benefits of combined hook and screw methods are not clear. Thus, the choice of which implant to use in a given case is often been based solely on a surgeon’s experience rater than on the biomechanical features and advantages of each device. The aim of this study was to measure the pullout strength of pedicle screws versus lamina hooks and combinations of screws and lamina hooks in porcine thoracic spine to simulate the surgical correction techniques used to treat scoliosis.

## Methods

**Figure 1:**
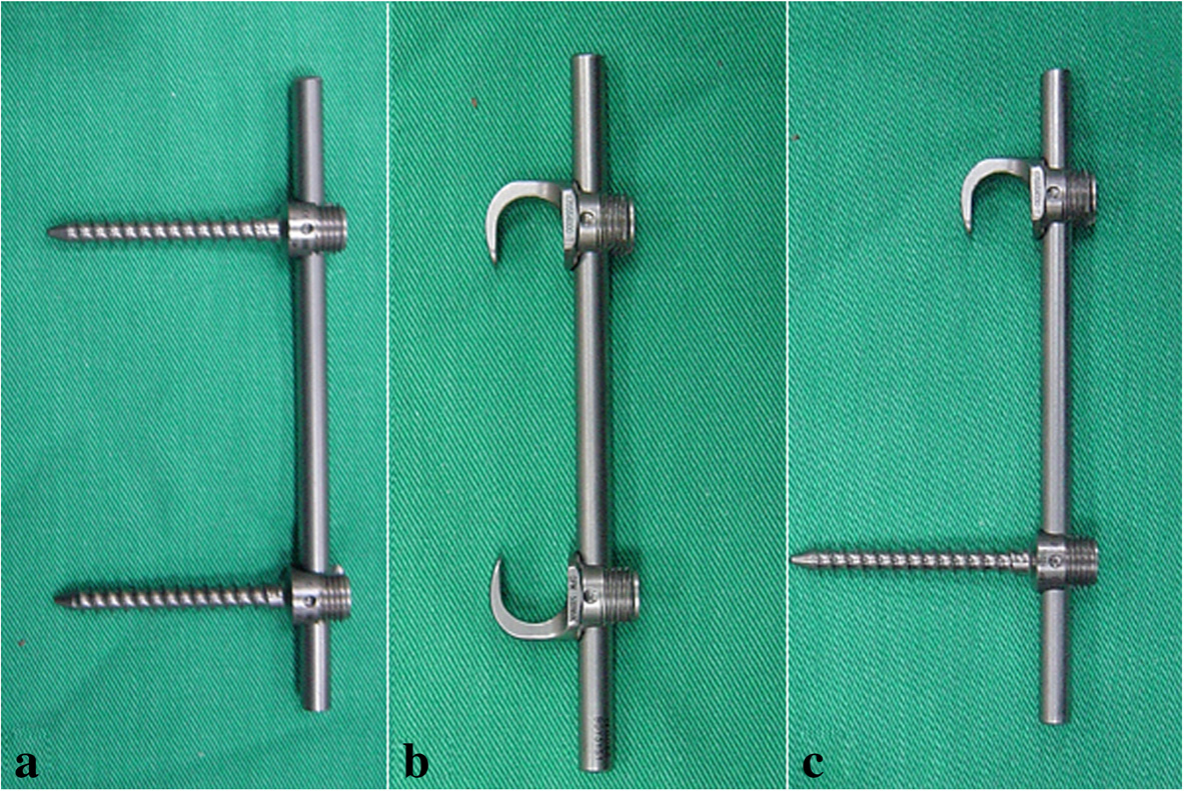
Photographs of (a) the pedicle screws, (b) the lamina hooks and (c) the combination of hooks and screws.

**Figure 2:**
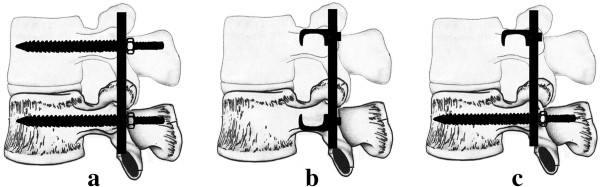
**Schematics showing the three combinations of screws and hooks instrumented in one spine motion unit. (a)** Pedicle screw, **(b)** Lamina hook, and **(c)** Screw-lamina hook.

**Figure 3:**
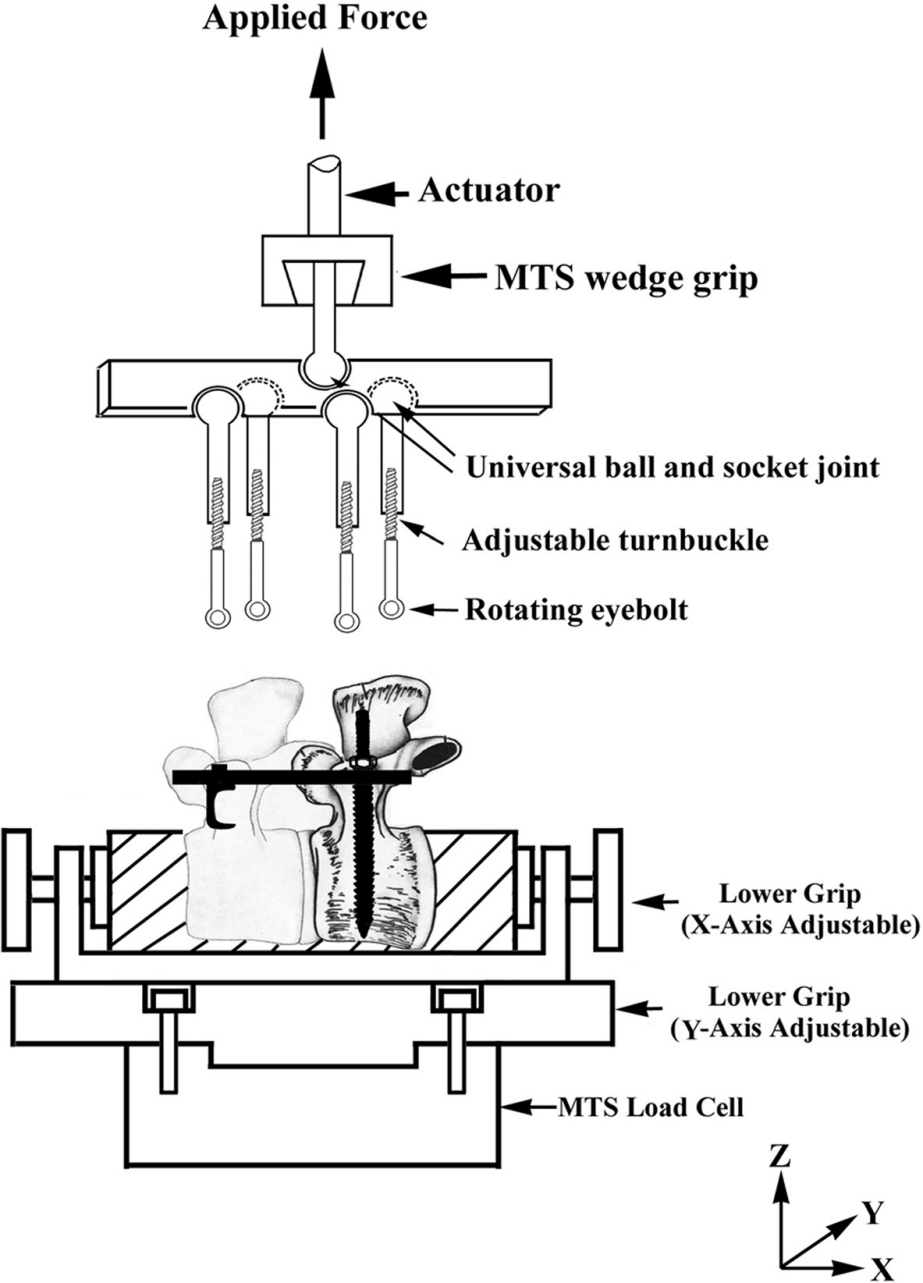
**Schematic showing the experimental setup of pullout test.** The force was applied in the direction normal to the rods.

This study was approved by the committee of National Science Council of Taiwan. All experiments conformed to the regulations for the care and use of animals. The usage of the porcine spines was in accordance with the guidelines of replacement, reduction and refinement. Thirty-six fresh lower thoracic porcine spines were assigned to three groups (12 per group) according to their fixation configuration: (1) pedicle screws, (2) lamina hooks and (3) combination of screws and lamina hooks. In the screw group (1), pedicle screws were inserted into the neighboring vertebral bodies of one spine motion unit. In the hook group (2), a claw configuration composed of two hooks directed toward each other was used. In the combination group (3), the hooks were placed at the proximal vertebrae, while the screws were inserted into the distal vertebrae. The constructs were connected by rods. Figure 1 shows the pedicle screws, lamina hooks and rods (Mossmiami, Depuy, USA) used in the current study. Figure 2 shows the three different combinations of screws and hooks used for spinal instrumentation.Implantation of the fixation screws was carried out under direct visualization. Radiological examinations were performed at the same time to confirm the depths and positions of the implanted screws in all of the specimens. An angled jig device was used to ensure accurate placement of the fixation screws. Axial pullout tests in a backward direction normal to that of the rods were performed using a material testing system (Bionix 858, MTS Corp., MN, USA). Custom-designed grips were secured to the actuator on the MTS machine. The grips consisted of universal ball-and-socket joints connected to steel plates. The plates had four variable attachment holes to accommodate specimens of different dimensions, along with four shoulder ball-and-socket joints lead to the four adjustable steel turnbuckles. The turnbuckles had rotating eyebolts on each end, which attached directly to the four exposed ends of the spinal rods extending from each of the constructs. The anterior portions of both segments of the spinal constructs were horizontally embedded in low-melting point Zn alloy (Figure 3). After the specimens were positioned, external forces were applied at a constant loading rate of 20 N per second. The relationship between the force and displacement was recorded at an increment of 50 N by the MTS Teststar-II software. To evaluate the effects of the various combinations of screws and hooks on the stability of the spinal constructs, the magnitude of the force at failure for each individual specimen was selected for comparison.

## Results

**Figure 4:**
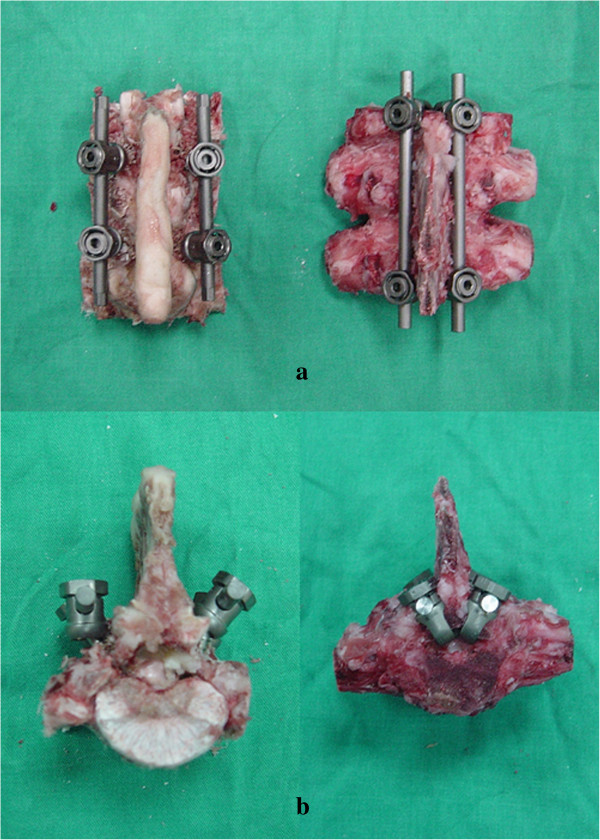
**Spinal constructs instrumented with pedicle screws (left) and a combination of screws and hooks (right). (a)** Posterior view, **(b)** Superior view.

**Figure 5:**
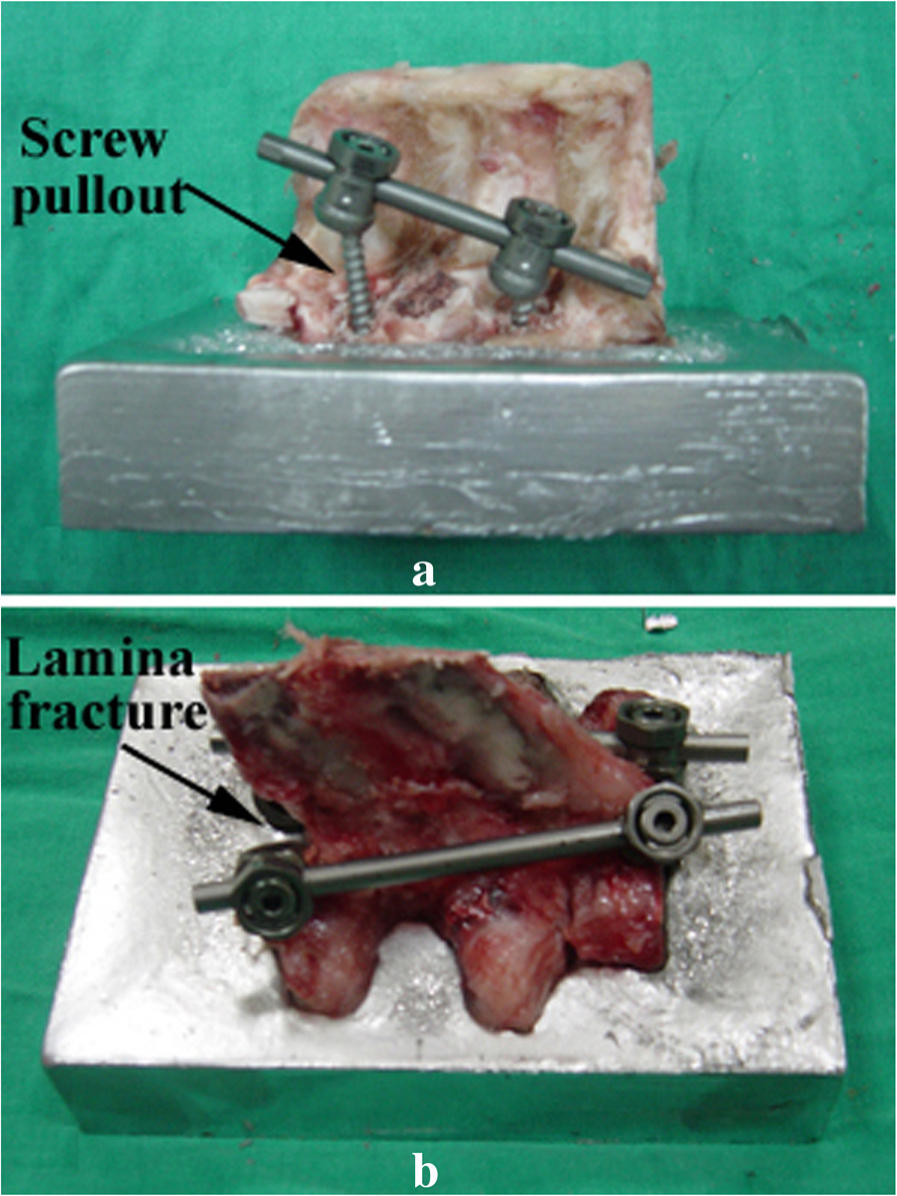
**The spinal constructs after the pullout tests. (a)** Spinal construct with pedicle screw instrumentations. The screws were pulled out without failure of the spinal construct. **(b)** Spinal construct with a combination of pedicle screws and lamina hooks. The spinal construct failed due to lamina fracture.

**Figure 6:**
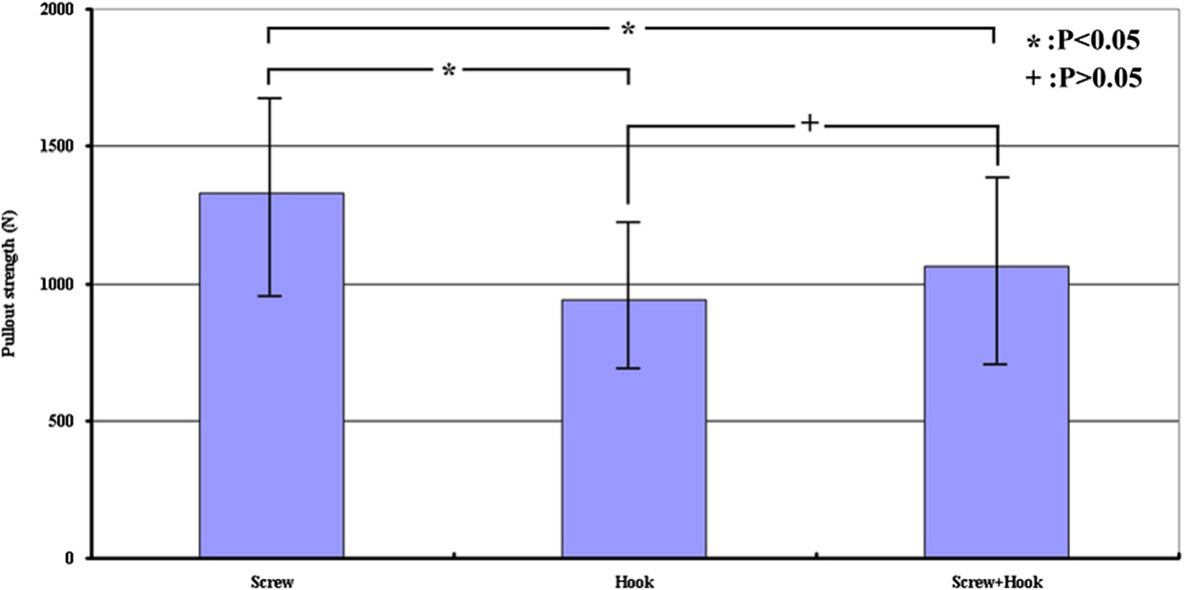
**Pullout force for the screw, hook and combination groups.** The pullout force for the screw group was significantly greater than for the hook and combination groups (p < 0.05). However, no significant difference was found between the hook and combination groups (p > 0.05).

The spinal constructs instrumented with the screws and with the combinations of screws and hooks are shown in Figure 4. The corresponding specimens after the pullout test are shown in Figure 5. All of the specimens were examined after testing. The destruction of vertebra in the combination group was considerably more severe than in the screw group (Figure 5). In the screw group, the screws were pulled out without destruction of the spine. In the hook and combination groups, however, the spinal construct failed due to fracture of the lamina. There was no screw or hook breakage throughout the testing. The fixation of specimen on the embedding alloy site was well maintained from the pretest condition (Figure 5).The maximum mean value of pullout force for the screw, hook and combination groups were 1,329 ± 381 N, 943 ± 259 N and 1,065 ± 297 N, respectively (Figure 6). The maximum pullout force of the pedicle screw group was 1.41 times higher than the hook group; whereas the pedicle screw group was 1.25 times higher than the combination group. These results indicated that the pullout force for the pedicle screw group was significantly greater than the other groups (p < 0.05). However, no significant differences were observed between the hook and combination groups (p > 0.05).

## Discussion

Because of the relatively early application of hooks for treating scoliosis, most surgeons are familiar with this technique. There are standard size hooks available for adolescent patients and miniature hooks available for small children (body weight < 30 kg). However, there are potential disadvantages associated with hook systems [12-14]. First, the tip of the lamina hook has to enter the neural canal, increasing the potential for injury to the spinal cord. Second, hooks engage only the posterior elements instead of engaging both the anterior and medial columns. Pedicle screws are more commonly used in the lumbar spine than in the thoracic spine. Placement of thoracic pedicle screws is an exact and demanding technique [15,16]. Surrounding the thoracic spine, there are many vital structures, including the trachea, aorta, segmental vessels, anterior vena cava, lung and spinal cord. Dr. Suk reported the use of thoracic pedicle screws to treat thoracic scoliosis in 1991 [11]. That report demonstrated improved radiographic results in patients treated with screw versus hook.

Spinal biomechanics have been studied in several ways. Using a long segment construct to study the biomechanics of implants is time consuming and impractical. To conquer this problem, it is reasonable to use a motion unit to study spinal biomechanics. The previous literature has examined the pullout behavior of pedicles, which were loaded by tension during flexion [17,18]. This situation can be exacerbated in long instrumentation constructs, where large axial pullout forces are generated due to cantilever bending at the proximal levels during forward flexion [17,18]. Our results indicated that the pullout force for pedicle screws was significantly greater than for hook and combinations of screws and hooks (p < 0.05). No significant difference was observed between the hook and combination groups (p > 0.05). Several studies have described the mechanical roles of pedicle screws and lamina hooks in spinal posterior instrumentation [19-21]. However, the anchoring strength for combinations of screws and hooks was not considered. Liljenqvist et al. [19] investigated the axial pullout strength of pedicle screws versus laminar hooks in the thoracic spine with respect to the surgical correction techniques used to treat scoliosis. Their results indicated that the average pullout strength of pedicle screws was significant higher than for laminar hooks. These observations were consistent with our results. Murakami et al. [20] compared fixation in the thoracolumbar spine using pedicle screws combined with infra-lamina hooks (at both the cranial and caudal ends of the same vertebrae) with fixation using pedicle screws alone. They concluded that a pedicle screw combined with an infra-lamina hook offers significantly greater strength compared to a pedicle screw alone. In osteoporotic vertebrae, the bone quality of the lamina is better than the bone quality in the vertebral body. Therefore, the bone mineral density might be an important consideration in the selection of screws or hooks. Hackenberg et. al [21] studied the fixation strength of pedicle screws versus pedicle and laminar hooks in the thoracic spine in relation to the bone mineral density. They concluded that pedicle screws may be beneficial for thoracic spine instrumentation because they offer significantly more resistant to axial and tangential loads than pedicle and lamina hooks on the condition that bone mineral density is higher than 100 mg hydroxyapatite/mL. However, the difference is not significant if bone mineral density is lower than 100 mg hydroxyapatite/mL.

Our study is an *in vitro* analysis of specimens prepared in a laboratory environment, which does not necessarily represent clinical circumstances. There are several limitations to our study. First, we used porcine spines instead of human cadaveric spines. Although the physiological structures are somewhat different from those of human cadaveric spines, animal spines are the most convenient choice for performing the experiments when human cadaveric spines cannot be accessed. Numerous studies [22,23] have been performed to evaluate the biomechanical behavior of spinal columns. These studies typically use porcine spines as models for the human spine. Smit’s report [23] showed similarities in the mechanics of quadruped and human spines. He concluded that a quadruped can serve as a valuable model for studying the human spine in spite of its horizontal position. Second, our work is limited to static loading (pullout) and does not consider other physiological loads. In actual physiological situations, the screw/bone and hook/bone interfaces are subjected to complex dynamic multi-directional loads. Consequently, the choice between a pedicle screw and a hook should not be determined solely based on the pullout strength. In practice, fixation devices with the ability to correct deformity, restore sagittal balance, prevent deterioration and influence adjacent segments should all be considered. Although the loading mode for pullout does not necessarily represent the actual physiological loading condition and may have great impact on the clinical relevance, the specimens used in this study were all prepared and tested in a uniform and reproducible manner. Thus, we believe that these results will provide useful information for orthopedic surgeons to assess spinal posterior instrumentation systems.

## Conclusions

The pedicle screw instrumentation achieves the maximal pullout strength for spinal posterior instrumentation. Based on this comparative study, surgeons should be better informed for selecting the optimal fixation method for the thoraco-lumbar spine before surgery.

## Competing interests

The authors declare that they have no competing interests.

## Authors’ contributions

CLT participated in the study design, data collection, statistical analyses and drafting of the manuscript. LHC, DML and MYL participated in the study design. PLL advised and assisted with drafting the manuscript. All of the authors read and approved of the final manuscript.

## Pre-publication history

The pre-publication history for this paper can be accessed here:

http://www.biomedcentral.com/1471-2474/15/197/prepub
